# Female Doctors

**Published:** 1883-02

**Authors:** 


### Female Doctors.

An Austrian lady, who has graduated as a doctor of medicine at Zurich, has been refused permission to practice by the Cultus Minister of Austria. He holds that the diplomas of foreign universities can only confer rights on those who would be eligible for degrees at Austrian universities; and as these do not admit women as students, his decision debars all women, however well qualified, from all those professions for which a university degree is required. It is expected an appeal will be made to the law courts.

Cockroaches, says a correspondent of *Land and Water*, who has lived with them in all the five quarters of the globe, will not touch bookbinding varnish with a mixture of 1 part copal varnish and 2 parts oil of turpentine. With a large brush paint this over the cloth binding, and let the book stand to dry. Unfortunately, it cannot be applied to the edges.

